# New Routes and Opportunities for Modular Construction of Particulate Vaccines: Stick, Click, and Glue

**DOI:** 10.3389/fimmu.2018.01432

**Published:** 2018-06-26

**Authors:** Karl D. Brune, Mark Howarth

**Affiliations:** ^1^Department of Bioengineering, Imperial College London, London, United Kingdom; ^2^Department of Biochemistry, University of Oxford, Oxford, United Kingdom

**Keywords:** vaccinology, virus-like particle, bioconjugation, malaria, synthetic biology, click chemistry, SpyCatcher

## Abstract

Vaccines based on virus-like particles (VLPs) can induce potent B cell responses. Some non-chimeric VLP-based vaccines are highly successful licensed products (e.g., hepatitis B surface antigen VLPs as a hepatitis B virus vaccine). Chimeric VLPs are designed to take advantage of the VLP framework by decorating the VLP with a different antigen. Despite decades of effort, there have been few licensed chimeric VLP vaccines. Classic approaches to create chimeric VLPs are either genetic fusion or chemical conjugation, using cross-linkers from lysine on the VLP to cysteine on the antigen. We describe the principles that make these classic approaches challenging, in particular for complex, full-length antigens bearing multiple post-translational modifications. We then review recent advances in conjugation approaches for protein-based non-enveloped VLPs or nanoparticles, to overcome such challenges. This includes the use of strong non-covalent assembly methods (stick), unnatural amino acids for bio-orthogonal chemistry (click), and spontaneous isopeptide bond formation by SpyTag/SpyCatcher (glue). Existing applications of these methods are outlined and we critically consider the key practical issues, with particular insight on Tag/Catcher plug-and-display decoration. Finally, we highlight the potential for modular particle decoration to accelerate vaccine generation and prepare for pandemic threats in human and veterinary realms.

## Introduction: The Importance and Challenge of Nanoparticle Vaccines

**Figure 1:**
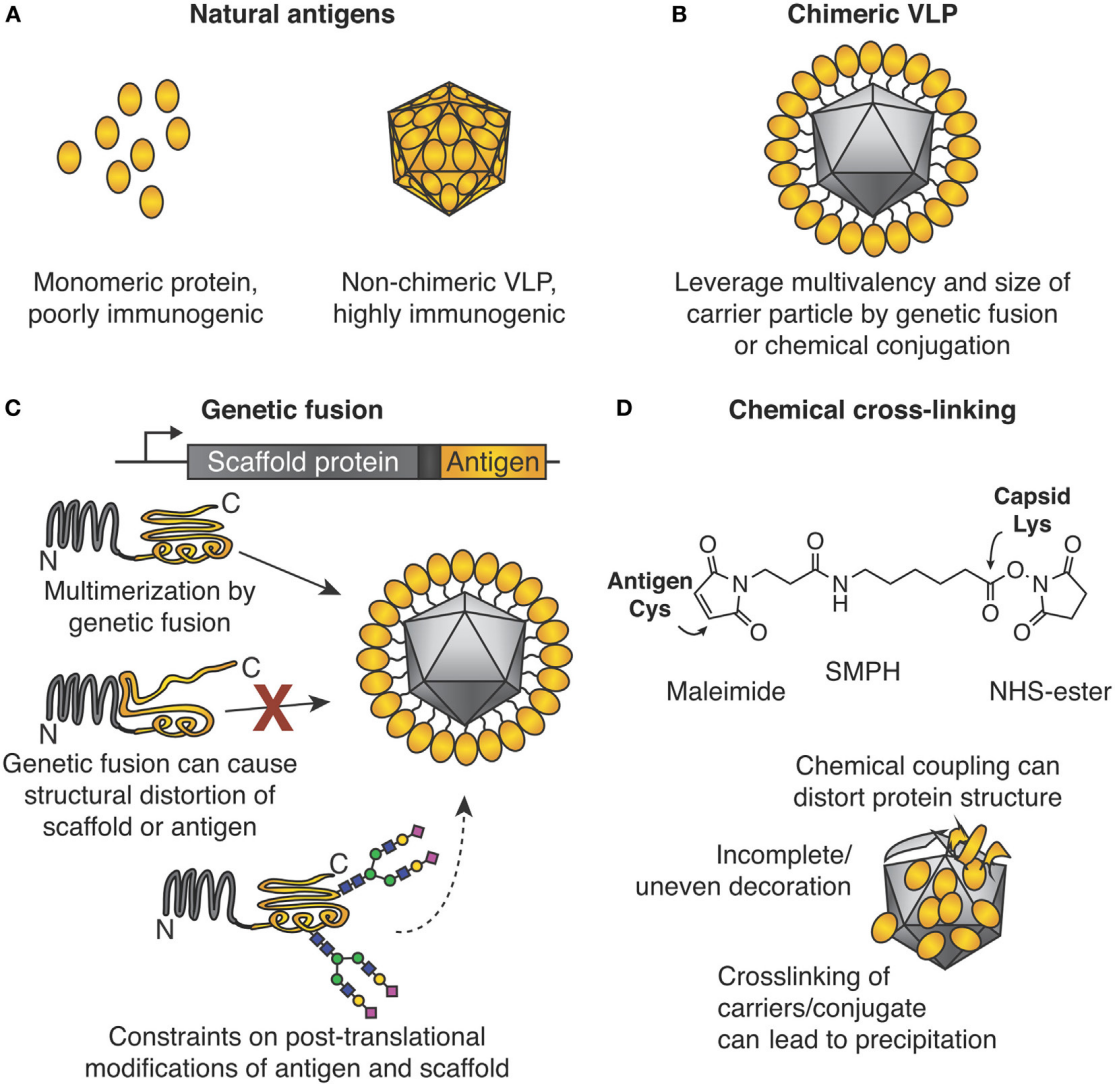
Chimeric virus-like particles (VLPs) and classic approaches for their decoration. **(A)** Importance of the particulate state for immunogenicity. **(B)** Chimeric VLPs leverage the multimeric nature of a scaffold for increased immunogenicity. **(C)** Genetic fusion for chimeric VLP assembly. The gene encoding an antigen of interest is fused to the gene encoding the multimerizing protein. Self-assembly leads to multimerization and ordered display of the antigen. Problems encountered may be (i) structural distortion of antigen or scaffold, which may lead to failed VLP assembly or induction of ineffective antibodies, or (ii) post-translational modification by the host may not be ideal for both antigen and multimerization platform. **(D)** Chemical cross-linking for chimeric VLP assembly. Side chains on the antigen and VLP are connected by a cross-linker, e.g., SMPH [succinimidyl 6-(β-maleimidopropionamido)hexanoate]. Problems encountered can be (i) distortion of structure of antigen or scaffold from uncontrolled conjugation, (ii) uneven decoration of VLP with antigen, and (iii) inter-particle cross-linking and subsequent impaired solubility.

The medical and commercial success of virus-like particle (VLP) vaccines against hepatitis B virus (HBV, vaccine ~22 nm in size) and human papillomavirus (HPV, vaccine ~55 nm in size) is attributed to their particulate nature (1, 2). The medical significance of VLPs is highlighted by the Nobel Prize in 1976 to Baruch Blumberg for work on HBV and identifying VLPs. Also, the 2017 Lasker Award in Clinical Medical Research went to Douglas Lowy and John Schiller for HPV vaccine development. These vaccines are non-chimeric, i.e., HBV VLPs protect against HBV infection (Figure 1A). The advent of genetic engineering allowed genetic fusion of a coat protein from one VLP to a protein antigen derived from a different pathogen (Figures 1B,C). Such a chimeric VLP acts as a scaffold to gain the benefits of multimerization. Lacking pathogenic nucleic acids, such vaccines show an excellent safety profile, although the immunogenicity may be less than live-attenuated vaccines (3).

Reasons for the potency of particulate vaccines have been reviewed (1, 4). Briefly, particulate vaccines mimic the size (>20 nm) and structural organization of viruses. The particulate vaccine efficiently enters lymphatic vessels and drains to lymph nodes (1). Here, the high-level B cell receptor cross-linking, caused by the repetitive high-antigen density on the particulate, can directly activate B cells without T cell help (4). In addition, IgM and complement interactions with the particulate vaccine may result in increased follicular dendritic cell (FDC) uptake of the particle (4). Increased FDC uptake then increases presentation of antigens in the lymph node, causing further B cell induction (2, 4). VLPs can also induce cytotoxic T cell responses *via* major histocompatibility complex (MHC) cross-presentation (4). Antigen-processing and cross-presentation have been reviewed elsewhere in detail (5). Certain VLPs have encapsulated nucleic acids, which can serve as toll-like receptor (TLR) ligands for cytokine signaling and further enhance the immune response (1, 4). Hereafter, we will refer to VLPs and other nanoparticles collectively as virus-like nanoparticles (VLNPs).

## The Difficulty of Installing Complex Antigens on VLNPs

To capitalize on the above advantages of particulate display, much effort has been directed to impart these advantages on monomeric or oligomeric antigens.

### Genetic Fusion and Its Challenges

In many areas of molecular biology, genetic fusion of two proteins is robust, and the first construct may be expected to retain the desired functions. For example, GFP fusions have been performed genome wide (6). For reasons discussed below, fusion of pathogen-derived antigens to VLP monomers for multimerization has not been as successful (Figure 1C).

#### The Termini of Coat Proteins Are Often Important for VLP Assembly

For many VLPs, one or both termini of the coat protein subunit are involved in inter-subunit interactions, e.g., Qβ or hepatitis B core antigen (7, 8). Termini at interfaces may impart stability, since the termini are the part of the protein most likely to flex. Fusion of a new protein at such a terminus is likely to destabilize the VLP. Alternatively, a terminus may face the inside of the VLP, so fused antigens are less likely to induce antibodies. Insertion in a loop of the capsid subunit may be tolerated for some short peptides, but insertion of a protein antigen in a loop of a capsid subunit will often disrupt the fold of the capsid and/or protein antigen (8, 9).

#### VLP Assembly Is Often Metastable

In natural infection, the virus capsid should not assemble as soon as the protein is made: assembly should be synchronized to packaging, e.g., of viral nucleic acid (10). Therefore, even though many VLPs are highly stable once assembled, small changes to the VLP (such as fusion) can cause major interference in the assembly pathway (11).

#### Cooperative VLP Assembly Means That Errors Propagate

If 5% of a monomeric protein is misfolded, that fraction can typically be purified away without consequence. If 5% of a VLP subunit is misfolded, those defective subunits may dock on to partly formed VLPs and prevent completion of the VLP assembly. Therefore, much greater than 5% malformed VLPs may result, giving dominant negative cooperativity. Folding of chimeric VLPs is highly sensitive to errors, e.g., from misfolding, mistranslation, incomplete post-translational modification, or unexpected interaction with other cellular proteins (11). Unmodified viral coat proteins have faced evolutionary pressure over many generations to fold and assemble optimally, so that such challenges have been addressed.

#### The Complexity of the Antigen

##### Multimeric State of the Antigen

Some antigens of interest are monomeric, but many are oligomeric. Viral glycoproteins are frequently trimeric (e.g., HIV gp120, influenza hemagglutinin). Given that many VLPs are icosahedral, the symmetry at the fusion site should match the symmetry of the intended fusion. Other important antigens are heteromultimeric, such as in herpesviruses.

##### Post-Translational Modification of the Antigen

A wide variety of VLNP scaffolds have been developed based on viruses and multimeric protein complexes from bacteria, insects, plants, and mammals. The merits of each VLNP expression system have been reviewed (12–14). However, the optimal expression host for a specific VLNP may not be optimal for expressing the specific target antigen. Phage-derived VLPs can be expressed at gram per liter scale in *Escherichia coli* (13). However, expression in *E. coli* does not typically allow for correct formation of disulfide bonds or complex glycosylation (15). Plant expression provides high yield at low production cost and improved disulfide formation, but may differ in glycosylation of antigens (14). Mammalian expression is generally considered the best platform for folding and post-translational modification of complex proteins. However, mammalian expression is the most expensive approach (16) and also susceptible to passenger viruses (e.g., simian vacuolating virus 40 or porcine circovirus-1), requiring stringent quality-control monitoring (16).

##### Antigens May Have Multiple Conformations

Often target antigens need to convert between conformations in their functional role, e.g., pre-fusion, fusion-active, and post-fusion conformations of viral glycoproteins (17). In some cases, display of the wrong conformation on the VLNP is not just neutral but harmful, as for respiratory syncytial virus vaccination (18). Sometimes, instability of antigen structure may have evolved to reduce the stability of antibody complexes (19).

##### Antigen Sequence Variability

Annual strains of influenza are a regular reminder that vaccine development must keep pace with a changing genetic landscape of pathogens (17, 20). HIV is one of the most variable human pathogens; major advances have been made in designing an HIV gp120 variant with a stabilized disease-relevant conformation to generate broadly neutralizing antibodies (17, 21).

##### Enhancing Genetic Fusion to VLPs

Various innovative approaches have been taken to overcome the above challenges for genetic fusion to VLPs. Fusion to ferritin at its threefold axis has enabled display of trimeric viral glycoproteins (22). Genetic dissection of the hepatitis B core antigen generated an exposed terminus, which was better able to display fusions on the VLP (SplitCore HBcAg) (8). However, sometimes compromises in genetically fused VLPs have decreased the chances of long-term success: if full domains cannot be genetically fused, fusion of VLPs to short peptides has often been tried or antigens were genetically dissected and distributed across the carrier (23, 24). Such peptide-decorated VLPs can generate antibodies that cross-react with the parent protein. However, compared with full domains, peptides on VLPs often decrease the proteolytic stability and solubility of the assembled VLP, as well as reducing the titer of neutralizing antibodies (25, 26). Also, genetic fusion of peptides to VLPs can sometimes cause toxicity to the expressing cells (27).

### Modular Vaccine Assembly

**Figure 2:**
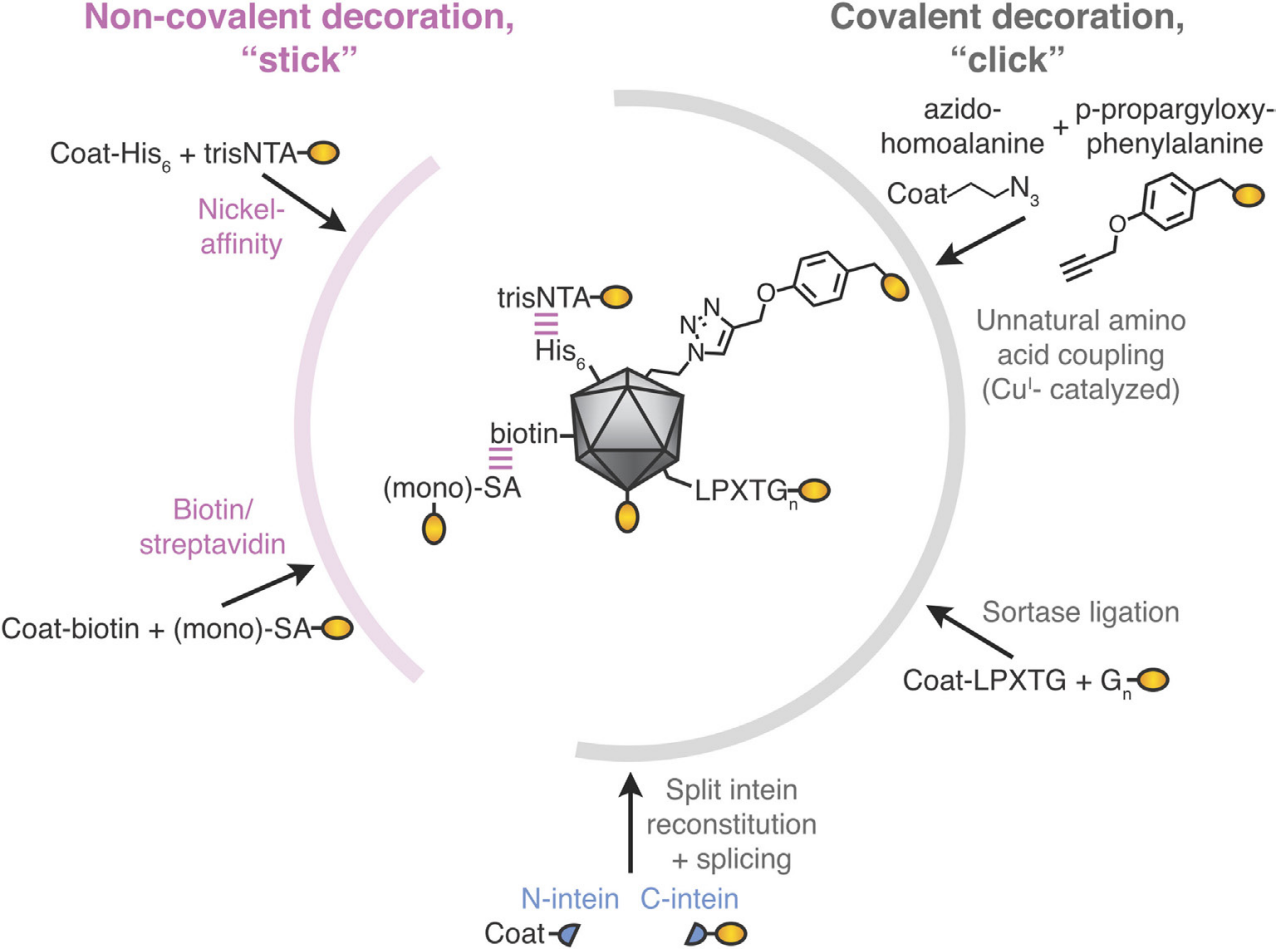
Stick or click approaches for decoration of virus-like particle (VLP) vaccines. Non-covalent decoration of VLPs is represented by nickel-affinity or biotin/streptavidin decoration (pink). Covalent decoration of VLPs is represented in gray. We illustrate unnatural amino acid incorporation *via* global incorporation of azidohomoalanine in the VLP, sortase ligation, or split intein trans-splicing. The antigen is represented in gold; not to scale.

Modular VLNP assembly refers to generating a VLNP-based vaccine in more than one stage. In particular, the VLNPs and antigen are generated separately and then coupled (Figures 1D and 2). Compared with genetic fusion, modular VLNP assembly adds an extra step, so in theory is more complex. In practice, modular assembly can side-step many of the challenges of genetic fusion. The VLNP and antigen can be generated in different systems, choosing whatever gives the optimal yield, conformation, and post-translational modification for each.

The difficulty of manufacturing a vaccine is a major factor in the overall vaccine cost (16). The Bill and Melinda Gates Foundation have set a target of US $0.15 per human vaccine dose, while even lower costs may be needed for veterinary and food-safety vaccines (28, 29). Mass production of an abundant, stable, and easy-to-conjugate VLNP scaffold could accelerate vaccine generation. Such a nanoparticle scaffold could also allow production costs to be shared across multiple antigens and diseases (30). Quickly and reliably multimerizing libraries of pathogen-derived antigens should speed up development of vaccines against the most challenging diseases, such as tuberculosis or malaria (31, 32).

Modular assembly is also attractive because personalized vaccines and pandemic threats will require an ever shorter time from development to manufacturing (33, 34). Pre-production of modular scaffold particles, that only need to be mixed with conjugation-enabled antigen or good manufacturing practice (GMP)-grade synthetic peptide, may be helpful for pandemic preparedness (35). As an indication of how governments prepare for pandemics, 40 million doses of a drug, oseltamivir, against influenza were purchased by the UK government (UK population ~66 million people) (36). UNICEF maintains 2 million doses of the oral cholera vaccine (37), while the World Health Organization has 35 million doses of smallpox vaccine stockpiled (38).

### Classic Approaches to Modular VLNP Decoration

What we term the classic approach to modular VLNP decoration is the use of cross-linkers to react with nucleophilic amino acid side chains on the VLNP and the antigen (Figure 1D). The most common reaction is acylation of an amino group from lysine or the N-terminus on the VLNP by the *N*-hydroxy succinimide arm of the cross-linker. This is followed by alkylation of a cysteine on the antigen by the maleimide arm of the cross-linker. Cysteine is rare on the surface of proteins, so often a single exposed cysteine can be introduced by site-directed mutagenesis (39, 40). VLP platforms for classic chemical coupling include the bacteriophage Qβ and tobacco mosaic virus and have entered a number of clinical trials, including for diabetes and addiction (41, 42). VLNP decoration with chemical cross-linkers has virtues of scalability and simplicity. Challenges arise when introducing a reactive cysteine interferes with formation of pre-existing disulfide bonds in the antigen (39). Since there are multiple lysines on the surface of the VLNP, coupling is usually heterogeneous and difficult to predict or analyze (Figure 1D). Less commonly, carboxylic acid residues are activated and then reacted with amines (43). It has been suggested that non-uniform coupling of antigen to VLNP may result in sub-optimal immunogenicity (see Catcher/Tag Technology for VLNP B Cell Vaccines).

## Latest Approaches in Post-Assembly Decoration of Particles

Chemical conjugation approaches to proteins in general have been reviewed elsewhere (44). Here, we specifically consider approaches used to decorate VLNPs and discuss their strengths and limitations, in contrast to the classic approaches above.

### Non-Covalent VLNP Decoration

#### His-Tag/Ni-NTA Affinity

The His-tag is the most commonly employed peptide motif for recombinant protein purification. Exploiting the interaction between a His-tag and nickel-loaded tris-nitrilotriacetic acid (trisNTA), the Hytönen lab decorated norovirus VLPs (Figure 2) (45). trisNTA was chemically linked to dye, vesicular stomatitis virus peptide or biotin. The His-tag binds to Ni-NTA with moderate affinity, which is reduced at lower pH because of its increased His protonation (46). Hence, re-shuffling of VLP assemblies may be an issue during storage or in the lower pH endosomes after vaccine injection.

#### Biotin–Avidin Affinity

To leverage one of the strongest non-covalent interactions, VLNPs may be biotinylated chemically or using biotin ligase. Biotinylated VLNPs are subsequently recognized by a member of the avidin family (Figure 2). Avidin and streptavidin are both tetrameric, which may cause challenges with VLNP cross-linking. However, one can use monovalent streptavidin (four subunits but only one subunit binds biotin) or monomeric streptavidin (one subunit but lower biotin affinity) (47–50). Early studies only coupled short peptides to VLNPs (51, 52), but the approach has since been adopted for full-length proteins up to 85 kDa (49). Even though biotin’s interaction to avidin/streptavidin has a high-affinity, half-times for biotin-conjugate dissociation are on the scale of hours (53), so re-shuffling is expected upon storage.

#### Anthrax Toxin Receptor Decoration

Flock house virus coat protein was genetically fused to part of the human anthrax toxin receptor (ANTXR2). This fusion allows decoration with anthrax protective antigen, which binds with 170 pM dissociation constant (54). High titer neutralizing antibodies to the affinity-displayed protective antigen were observed after administration. No significant antibody response was observed against endogenous ANTXR2, indicating that this platform did not break self-tolerance (54).

### Novel Covalent VLNP Decoration Methods

Covalent reaction between the target antigen and particle brings the obvious advantage of stability. This includes not only stability during storage but also stability after injection, where the lower concentration and altered conditions (e.g., endosomal pH, shear force in the circulation) favor dissociation of non-covalently linked antigen from the particle. Covalent conjugation also facilitates analysis of decoration. A simple SDS-PAGE can quantify what proportion of particle subunits are coupled to antigen and how much non-covalently associated antigen remain (55). Covalent coupling methods are described below, compared in Table 1, and illustrated in Figure 2.

**Table 1:** Features of covalent protein–protein coupling methods.

Method	Terminus restriction	Redox dependence	Orthogonal pairs	Scar after coupling	Other features or challenges
Split intein	N-intein at C-terminus, C-intein at N-terminus	Yes, for Cys-dependent split inteins	Yes	0–3 aa	–

Sortase	C-terminal-LPXTGX and N-terminal oligoglycine/alanine	No	*S. aureus* and *S. pyogenes* enzymes	Small (7 aa)	Need high conc. of oligoglycine reactant; challenging to reach complete conjugation

Tag/Catcher	Tag or Catcher at N- or C-terminus or internal	No	SpyCatcher, SnoopCatcher	Catcher (≥84 aa) and Tag (12–13 aa) retained	High reaction yield

Electrostatic interaction lock	Should not be restricted	Reducing before reaction; oxidizing after reaction	In principle: several coiled-coil pairings known	Moderate (typically 18 aa)	Disulfides may reverse

Unnatural amino acid coupling	None	No	Yes	1 aa in Ag and 1 aa in virus-like particle	Cost/complexity of expression

#### Incorporation of Unnatural Amino Acids to Enable Click Chemistry

Several groups have decorated VLNPs by translationally introducing unnatural amino acids as uniquely reactive chemical handles. For example, the unnatural amino acid azidohomoalanine was incorporated into the coat proteins of MS2 or Qβ bacteriophage *via* cell-free protein synthesis (56). Using this global replacement strategy, >85% of methionines was replaced with azidohomoalanine in the coat protein of the VLP (Figure 2) (56). Alternatively, unnatural amino acids can be site-specifically incorporated when an amber stop codon in the mRNA is recognized by a tRNA incorporating the unnatural amino acid (56, 57). The most widely adopted unnatural amino acids utilize click chemistry, which refers to reactions of functional groups that occur rapidly, selectively and in high yield. The most commonly used click-chemistry reactions are alkynes with azide in the presence of Cu^I^ catalysis (44). Faster and catalyst-independent click reactions have also been developed (44). VLNP decoration using unnatural amino acids has the advantage of requiring minimal change (1 amino acid) to the antigen. Challenges for scaling up come from the cost and side reactions of the unnatural amino acid (58, 59). Also, assembly of a VLNP is cooperative and so a few percent of mistranslated or misfolded subunits may impair a majority of the resultant capsids (see Genetic Fusion and Its Challenges).

#### HaloTag

HaloTag, a modified haloalkane dehalogenase, binds irreversibly to haloalkane ligands. HaloTag has been used to decorate the 60-mer E2p from *Geobacillus stearothermophilus* with DNA strands and to decorate other nanoparticles with short peptides (60, 61). Strengths of this approach are that HaloTag does not require cofactors or post-translational modifications for its coupling reaction and HaloTag reacts specifically in cellular systems. A possible drawback is the 33-kDa size of HaloTag and the requirement to install a synthetic haloalkane ligand on the antigen or VLNP substrate protein (62).

#### SNAP-Tag

SNAP-Tag is a 20-kDa protein which binds irreversibly to alkyl-guanine ligands. SNAP-Tag has been used to conjugate single-chain variable fragments of antibodies (scFvs) to nanoparticles (63). SNAP-Tag has also been used to derivatize HIV-1 virions (64). These virions were capable of assembly and infection but displayed reduced replication (64).

#### Sortase

Sortase is an enzyme from Gram-positive bacteria that covalently joins proteins with a C-terminal LPXTGX motif to other proteins with an N-terminal oligoglycine motif (Figure 2) (65). Sortase A-mediated ligation was used to conjugate to a genetically installed LPETGG motif on the surface of HBV core antigen VLPs and has since been adopted to other carriers (8, 66). Decoration efficiencies can be high (90%), but may require 6- to 12-fold molar excess of antigen (up to 480 µM) (66). Sortase was used to decorate E2p nanoparticles with proteins up to 150 kDa, but yielded only sparsely decorated 60-mers (60, 67).

#### Split Inteins

Split inteins are a naturally occurring protein chemistry, where N- and C-terminal intein regions associate non-covalently and then splice each other out, leading to the attached fragments (exteins) being joined by a peptide bond (Figure 2) (68). Split inteins have subsequently been enhanced by rational design and evolution. Note that in the literature SpyTag/SpyCatcher (see Spontaneous Isopeptide Bond Formation) has sometimes been mistakenly referred to as a split intein. Split inteins have been used to generate a C-terminal thioester on a protein, with subsequent conjugation to flock house virus *via* native chemical ligation (54). Split inteins have also been used to functionalize adeno-associated virus particles with scFvs or DARPins, showing a coupling yield up to 10% (69). Some of the fastest split inteins operate *via* a reactive cysteine, which may be problematic with disulfide-containing antigens (see Classic Approaches to Modular VLNP Decoration). However, in other split inteins, the key reactive residue is serine or threonine (68).

#### Electrostatic Interaction Locks (EILs)

Electrostatic interactions have been utilized to align cysteine residues to form preferred disulfide bonds, yielding EILs (70). Attempts at locking larger proteins, such as antibody Fv fragments to viral coat protein suffered from <20% decoration efficiencies (71). More recent EIL-mediated peptide display on bovine papilloma virus was successful but reduced particle uniformity (9).

#### Spontaneous Isopeptide Bond Formation

**Figure 3:**
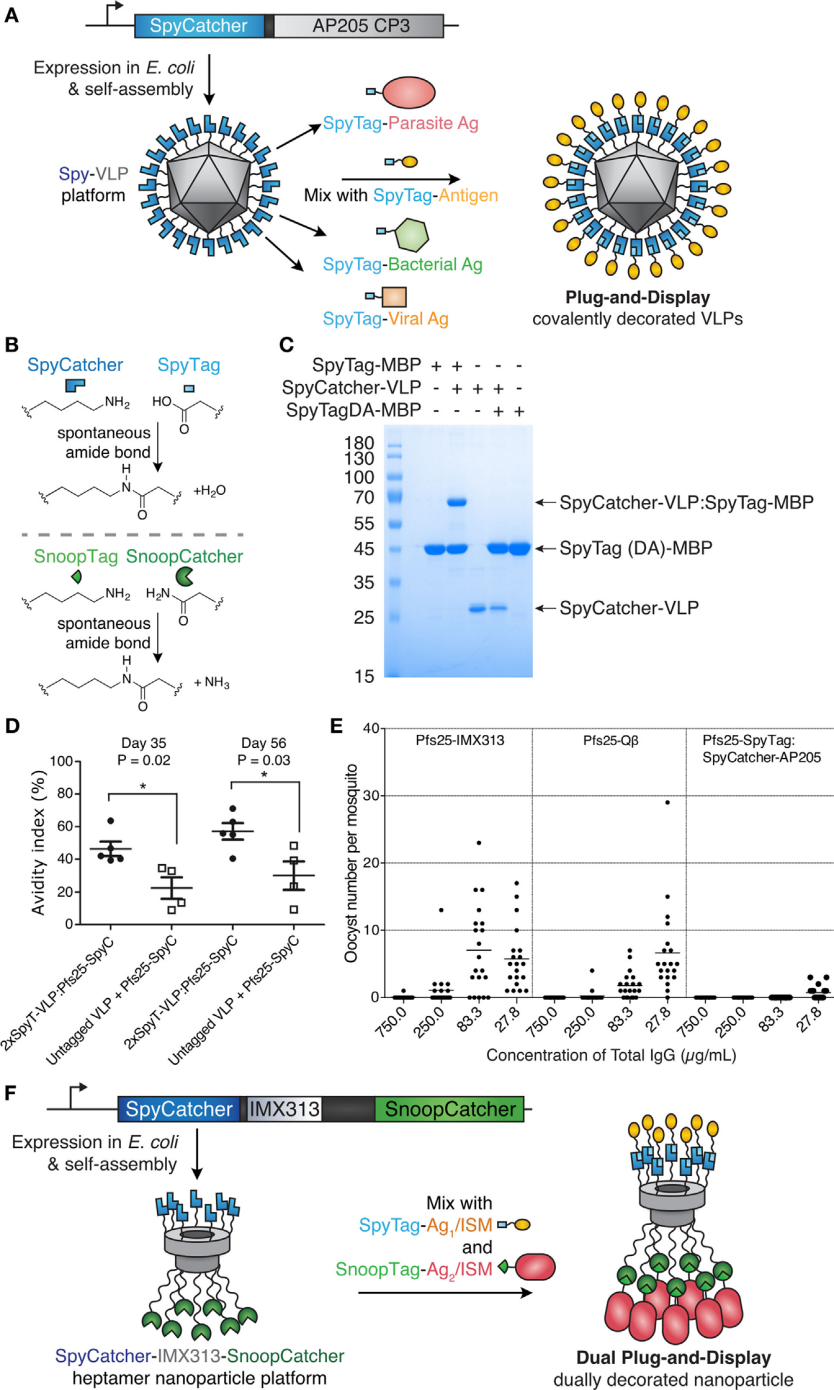
Plug-and-display virus-like particle (VLP) decoration. **(A)** Plug-and-display principle. SpyCatcher was genetically fused to the AP205 coat protein and expressed in *Escherichia coli*. Upon mixing, different SpyTag antigens form spontaneous isopeptide bonds, yielding decorated particles for immunization. **(B)** Catcher/Tag chemistry. SpyCatcher/SpyTag side chains form a covalent bond with release of water. Reaction of SnoopCatcher with SnoopTag yields a covalent bond and release of ammonia. **(C)** Quantitative coupling of Spy-VLPs with antigen. SpyCatcher-VLPs were incubated with SpyTag-maltose-binding protein (MBP) or the negative control SpyTagDA-MBP, before SDS-PAGE with Coomassie staining. Data from Ref. (55) with permission. **(D)** Improved antibody quality by Spy-VLP display. Antibody avidity (based on resistance to 8M urea) was assessed on days 35 and 56 in serum from mice immunized with Pfs25-SpyCatcher and untagged or dual SpyTag-VLPs. Data from Ref. (75) with permission. **(E)** Malaria transmission blocking with Spy-VLPs. Pfs25-IMX313 is a genetically fused nanoparticle. Qβ-Pfs25 is a chemically decorated VLP. Pfs25-SpyTag:SpyCatcher-AP205 is a plug-and-display conjugate. Total IgG, purified from pooled serum after immunization, was analyzed by standard membrane feeding assay. Data points represent oocysts in a mosquito, and the lines show the arithmetic mean. Data from Ref. (39) with permission. **(F)** Dual plug-and-display. Expression in *E. coli* and spontaneous multimerization yields SpyCatcher-IMX-SnoopCatcher nanoparticles. SpyTag linked to antigen 1 (Ag1) or an immunostimulatory molecule (ISM) react with SpyCatcher, while the other nanoparticle face is decorated by SnoopTag/SnoopCatcher reaction.

Catcher/Tag technology was engineered from certain cell-surface proteins of Gram-positive bacteria, which form autocatalytic intramolecular isopeptide bonds (72). Such isopeptide bonds can confer resilience to chemical (pH 2), thermal (>100°C), and mechanical (>1,000 pN) challenges (73). The SpyTag peptide (13 amino acids) forms a spontaneous isopeptide bond upon mixing with the SpyCatcher protein (116 amino acids) (Figure 3) (73). SpyTag/SpyCatcher reaction can proceed to >99% efficiency on VLNPs at modest excess (1.5-fold) (55, 73). SpyTag/SpyCatcher reacts under diverse conditions, such as pH 5–10 and 4–37°C, in the presence or absence of reducing agent (55, 73). Similarly, the SnoopTag peptide forms a spontaneous covalent bond to the SnoopCatcher protein and has no cross-reaction with SpyTag/SpyCatcher (74). Catchers and Tags react *via* side chains, so each partner can react independently of the location within a protein, i.e., either at the N- or C-terminus, or internally within a protein sequence (72). For many pathogen-derived antigens, there is good understanding where antibodies should bind for optimal neutralization (17, 19). Therefore, the ability to position SpyTag at various locations provides control in how the antigen will extend from SpyCatcher-linked VLNPs.

Given our greater expertise on Catcher/Tag VLNP decoration, we discuss the progress, opportunities, and limitations of this approach in more detail below.

## Adoption of Catcher/Tag Technology for Vaccine Assembly

### Catcher/Tag Technology for T Cell Vaccines and Cytotoxic Cancer Immunotherapy

The first application of Catcher/Tag technology toward immune modulation was the development of SpyAvidins—variants of streptavidin bearing either SpyTag or SpyCatcher on specific subunits of the tetramer. SpyAvidin-MHC eicosamers (bearing 20 streptavidin subunits) surpassed conventional MHC tetramers in stimulating T cell activation (76). In another study, SpyTag was fused at the C-terminus of anti-DEC205 scFv, to allow modular targeting of antigens to dendritic cells (DCs) (77). SpyCatcher-OVA8-TBEV-ED3 is a fusion protein that includes a CD8^+^ T cell epitope (OVA8, residues 257–264 from ovalbumin) and a B cell epitope (Tick-borne Encephalitis Virus ED3). Linking antigen to scFv *via* SpyTag/SpyCatcher allowed targeting of antigen to DEC205-positive DCs for cytotoxic T cell stimulation (77). The same DC-targeting platform subsequently induced potent cytotoxic T cell responses against the HPV oncogene E7, translating to therapeutic antitumor responses. Use of checkpoint inhibitors further enhanced the therapeutic effect (78).

### Catcher/Tag Technology for VLNP B Cell Vaccines

Brune et al. and Thrane et al. independently developed Spy-VLPs (Figure 3A) (55, 75). N-terminally truncated SpyCatcher (79) was fused to the coat protein of the bacteriophage AP205 (55). SpyCatcher-VLP reaction could proceed to quantitative yield (Figure 3C) (55). SpyCatcher-VLPs (~20–43 nm in size, undecorated) were efficiently decorated with SpyTagged complex antigens, such as two *Plasmodium falciparum* blood-stage CIDR antigens or the transmission-blocking ookinete antigen Pfs25 (55). Spy-VLPs induced high titers against Pfs25 after a single immunization (55). Spy-VLP-induced antibodies showed high avidity (Figure 3D) (75).

There is a shortage of side-by-side comparisons of vaccine platforms in the literature. As an initial test of different immunization platforms, mice were immunized with Pfs25 displayed on (i) SpyCatcher-AP205 VLPs using plug-and-display conjugation, (ii) Pfs25-VLPs from chemical decoration of Qβ, or (iii) genetic fusion of Pfs25 to the nanoparticle IMX313. Qβ display generated the highest anti-Pfs25 titer. However, SpyCatcher-VLP display gave the highest antibody quality, assessed by the ability of a given concentration of antibody to block malaria transmission to mosquitoes, using a standard membrane feeding assay (Figure 3E) (39, 55). Given the difficulty of varying one feature of a nanoassembly at a time, the molecular features determining the size and specificity of antibody responses need further investigation.

Thrane and co-workers demonstrated fusion of SpyTag to the N- and C-termini of the AP205 coat protein. Spy-VLPs (36–42 nm in diameter, undecorated) were conjugated to a range of antigens from diverse viral and bacterial pathogens (up to 118 kDa) (75). Spy-VLP display enabled breaking of tolerance against self-antigens (including PD-L1, CTLA-4, IL-5, and HER2) (75, 80). Immunization using SpyTag-AP205-SpyTag VLPs decorated with SpyCatcher-HER2 (extracellular domain) induced autoantibodies against human HER2. Four out of five mice were protected against a HER2-positive cancer challenge after prophylactic vaccination (80). Furthermore, the HER2-decorated Spy-VLPs (~66 nm in diameter, decorated) were also 100% effective in preventing the onset of mammary carcinomas in a mouse model expressing the Delta16 isoform of HER2, a splice variant more oncogenic than full-length HER2 (80). It will be interesting to explore the Spy-VLP approach against other cancer immunotherapy targets.

The range of antigens and platforms linked using Tag/Catcher at the time of submission is summarized in Table 2.

**Table 2:** Disease-related proteins assembled using Catcher/Tag technology.

Category	Antigen	Antigen expression	Platform	Platform expression	Reference
Self	Peptide-major histocompatibility complex class I multimers	*Escherichia coli*	Streptavidin-SpyTag, Streptavidin-SpyCatcher	*E. coli*	(76)

Cancer (Neo-self)	EGFRvIII-SpyTag	Chemically synthesized	ΔN1SpyCatcher-AP205	*E. coli*	(55)

Cancer (Neo-self)	SpyTag-Telomerase	Chemically synthesized	ΔN1SpyCatcher-AP205	*E. coli*	(55)

Cancer (HPV)	SpyCatcher-E7	*E. coli*	Anti-DEC205-SpyTag	Mammalian	(78)

Cancer (Breast)	SpyCatcher-HER2	Insect	SpyTag-AP205-SpyTag	*E. coli*	(80)

Malaria	SpyTag-CIDR	*E. coli*	ΔN1SpyCatcher-AP205	*E. coli*	(55)

Malaria	Pfs25-SpyTag	Mammalian	ΔN1SpyCatcher-AP205	*E. coli*	(39, 55)

Malaria	SpyCatcher-CIDR	Insect	SpyTag-AP205	*E. coli*	(75)

Malaria	CSP-SpyCatcher	Insect	SpyTag-AP205	*E. coli*	(75)

Malaria	Pfs25-SpyCatcher	*E. coli*	SpyTag-AP205-SpyTag	*E. coli*	(75)

Malaria	SpyTag-VAR2CSA	*E. coli*	SpyCatcher-AP205	*E. coli*	(75)

Malaria	CSP-SpyCatcher (Full-length 3d7 CSP)	Insect	SpyTag-AP205	*E. coli*	(81)

Malaria	Simultaneous display of: Pfs25-SpyTag, Pfs28-SnoopTag	Mammalian	ΔN1SpyCatcher-IMX-SnoopCatcher	*E. coli*	(82)

Malaria	SpyCatcher.R0.6C (GLURP fusion of partial Pfs45/48)	*Lactococcus lactis*	SpyTag-AP205-SpyTag	*E. coli*	(83)

Malaria	SpyCatcher-6C (fragment of Pfs45/48)	*L. lactis*	SpyTag-AP205-SpyTag	*E. coli*	(83)

Malaria	CSP-SpyCatcher conjugated to SpyTag-DBL1x-DBL2x-ID2a	Insect	N/A	N/A	(84)

Pneumococcal	SpyCatcher-(PspA α′-domain)-SnoopTag	*E. coli*	HbpD(Δd1)-SpyTag	*E. coli/Salmonella Typhimurium*	(24)

Pneumococcal	SpyCatcher-SP1690-SnoopTag	*E. coli*	HbpD(Δd1)-SpyTag	*E. coli/S. Typhimurium*	(24)

Pneumococcal	SpyCatcher-PsPα-SP1960-SnoopTag	*E. coli*	HbpD(Δd1)-SpyTag	*E. coli/S. Typhimurium*	(24)

Self	SpyCatcher-IL-5	Insect	SpyTag-AP205	*E. coli*	(75)

Self	SpyCatcher-HER2 extracellular domain	Insect	SpyTag-AP205	*E. coli*	(75)

Self	SpyCatcher-Survivin	Insect	SpyTag-AP205-SpyTag	*E. coli*	(75)

Self	SpyCatcher-IL-5	Insect	SpyTag-AP205-SpyTag	*E. coli*	(75)

Self	JoTag-transmembrane helices of GPCRs (C5aR)-InCatcher	*E. coli*	N/A	N/A	(85)

Self (heart disease)	PCSK9-SpyCatcher	Insect	SpyTag-AP205	*E. coli*	(75)

Self (T cell immunity)	CTLA-4-SpyTag	Insect	SpyCatcher-AP205	*E. coli*	(75)

Self (T cell immunity)	PD-L1-SpyTag	Insect	SpyCatcher-AP205	*E. coli*	(75)

Tick-borne encephalitis virus	SpyCatcher-OVA_8_-ED3	*E. coli*	Anti-DEC205-SpyTag	Mammalian	(77)

Tuberculosis	SpyCatcher-Ag85A	*E. coli*	SpyTag-AP205	*E. coli*	(75)

*N/A, not applicable*.

### Decoration of VLNP With More Than One Different Component

Decoration of VLNPs with multiple ligands is technically difficult, but may be important for protecting against different strains of a pathogen or even against distinct diseases (86). For classic approaches to VLNP decoration (see The Difficulty of Installing Complex Antigens on VLNPs), the challenge of dual display increases substantially, because of increased misfolding of larger genetic constructs and the difficulty in finding compatible chemistry without cross-linking particles. In one example, a cytokine (GM-CSF) and a scFv (each bearing alkynes) were simultaneously displayed on an azido-derivatized MS2 VLP, by carefully controlling ratios of both alkyne ligands (56). The resulting VLP may represent a range of slightly differently decorated particles with different immunological properties, since the precise location and spacing of multiple ligands cannot be controlled when a single reaction handle is used for attaching multiple proteins.

To avoid heterogeneity, it is paramount to identify conjugation strategies that are orthogonal to each other. Click chemistry and His-tag protein display, for example, have been combined on gold nanoparticles, enabling simultaneous display of influenza hemagglutinin and immunostimulatory flagellin (87). We set out to expand the Catcher/Tag VNLP approach to dually addressable Catcher/Tag protein nanoparticles (Figure 3B) (74). The multimerization domain IMX313 was recombinantly expressed with SpyCatcher at its N-terminus and SnoopCatcher at its C-terminus, yielding dually addressable protein NPs (~16 nm in diameter, undecorated) (Figure 3F). This VLNP allowed independent decoration of SpyCatcher with Pfs25-SpyTag and decoration of SnoopCatcher with Pfs28-SnoopTag. Immunization with this dually decorated VLNP yielded antibodies to both these *P. falciparum* transmission-blocking antigens, enhancing the response compared with monomeric antigen (82).

### Practical Aspects of Catcher/Tag Technology

#### Tag/Catcher-Fusion Effects on Antigen Expression

An important consideration for any vaccination platform is how easily the method can be adapted to different antigens. Ideally, it should be possible to express the modified protein with a yield and quality that is comparable to the untagged antigen. There are only a few fusion partners, such as glutathione-*S*-transferase and GFP, where solubility and expression data are available on thousands of fusions (6, 88). For SpyCatcher/Tag, these data are still limited but growing quickly (Table 2). There are promising signs that fusion of SpyTag (13 aa) to a protein often has little effect on its expression. SpyCatcher fusion, on the other hand, might be expected to cause more issues because of its bigger size (84–116 aa). Surprisingly, SpyCatcher fusion enhanced soluble expression in *E. coli* of the disulfide-rich Pfs25 from *P. falciparum* (75). Fusions to Catcher and Tag sequences have been expressed in various systems, including Gram-positive and Gram-negative bacteria (73, 89, 90), mammalian cells (73), insect cells (75), plant cells (91), and yeast (92).

#### Improving Vaccine Stability

The need for thermostable vaccines is widely recognized. Failure of the cold-chain has often led to wasting of vaccines or administering despite loss of activity (93). Important advances have been made by optimizing the engineering and chemistry of vaccine formation. However, the intrinsic stability of the protein components may also have profound effects on the magnitude and quality of the immune response (94, 95).

The dually addressable SpyCatcher-IMX-SnoopCatcher particles (Figure 3F) remained soluble after incubation at 99°C, while efficient Tag-antigen reaction was retained following incubation up to 60°C (82). Immobilization of enzymes onto potato virus X VLPs *via* SpyCatcher/SpyTag technology also suggests improved heat tolerance up to 50°C (91). Intramolecular cyclization *via* “sandwiching” enzymes using Tag/Catcher can impart high resilience to heat inactivation and even allow purification of an enzyme *via* boiling (89, 96). One could contemplate harnessing this resilience for purification and sterilization for cheap production of monomeric antigens (16, 29).

Similarly, cyclization of coxsackievirus B3 (CVB3) coat protein VP1 *via* split inteins averted irreversible heat aggregation, increased the proteolytic resilience, and reduced degradation *in vivo* (94). Upon immunization, circularized VP1 gave increased CVB3-specific antibody and cellular immune responses, improving protection against viral myocarditis (94).

## Limitations and What is Not yet Known for Catcher/Tag Technology

Here, we describe some key issues for modular decoration, moving from proof of principle toward real world application. We illustrate these challenges for SpyTag/SpyCatcher-based assembly, but many issues may be generalized to other decoration technologies.

### Structural Compatibility of Catcher/Tag-Antigen Fusions

Catcher/Tag decoration appears feasible for a range of non-enveloped VLPs (55, 75, 91, 97). Also, a wide range of antigens have now been displayed for immunization using SpyTag/SpyCatcher (Table 2). However, non-functional fusion proteins are much less likely to be published, so the literature is tilted toward functional fusion. In the future, it will be important to perform systematic studies on how the intrinsic multimeric state of an antigen affects VLNP display efficiency and the characteristics of the decorated particles. One may expect that antigens forming higher-order homomultimers would not be suitable for plug-and-display, because of the danger of cross-linking between particulates. However, IL-5 and survivin, typically dimeric (98, 99) have been successfully displayed on Spy-VLPs (75). It is possible that strategic placement of SpyCatcher/SpyTag close to multimerization interfaces may minimize particle cross-linking.

### Purification and Characterization of VLNPs

Yields for multimeric scaffolds of Catcher/Tag-VLPs (AP205 or IMX313) are still at the research scale of 1–20 mg/L using shaking-flask culture (55, 82, 83). Even though fermentation will increase biomass, it will be important to optimize these yields or identify other more scalable VLPs.

Molecules co-purifying with VLNPs are also an issue, when moving to clinical development. VLP platforms can package nucleic acids during assembly (100). Specific viral RNAs also act as scaffolds, guiding assembly of the viral capsid (10, 101). Intracellular nucleic acid detection is mediated particularly by the TLR family, e.g., TLR3 (dsRNA), TLR7 (ssRNA), and TLR9 (unmethylated CpGs in DNA) (100). AP205 VLPs contain 25–30 µg of host cell RNA per 100 µg of coat protein (102). Efficient removal of endotoxin from *E. coli*-expressed Spy-VLPs has been demonstrated, important for well-controlled comparison between immunogens (55). Endotoxin may be easily removed from Spy-VLPs, either by washing with Triton X-114 when the VLPs are immobilized on purification-resin or by phase separation (55).

Virus-like nanoparticle purity also depends on the yield of any coupling reactions. SpyTag/SpyCatcher in isolation gives a high yield of reaction (>99%) (Figure 3C) (74); the mechanism allows little possible side-reactions. Complete coupling of antigens to SpyCatcher-VLPs after 3 h was achieved in some cases, as assessed by SDS-PAGE using 1.5-fold molar excess of SpyTag-antigen over SpyCatcher-coat protein (55). In other cases, where there was a shorter spacer between the SpyCatcher and coat protein, coupling was less complete (75, 81, 83). Coupling may become slower on the crowded VLP surface, since already-coupled antigens sterically inhibit access of unattached antigens. SpyCatcher coupling may be optimized by exploring reaction conditions, in particular decreasing to pH 6 (55, 73). Faster variants (named SpyTag002/SpyCatcher002) have been engineered, which may allow faster VLNP coupling (103). In other cases, adjustment of the spacer between SpyCatcher/coat protein and SpyTag/antigen may be needed for large antigens. Alternative Catcher/Tag pairs have also been engineered that can cater to different pH-dependent conjugation optima, which may be more suitable for a particular demanding antigen or expression system (74, 85, 104–106).

### Does the Immune Response to the VLP Platform Help or Hinder Vaccination?

Chimeric VLPs such as RTS,S (the malaria vaccine based on HBsAg VLPs) will contain immunologically relevant sequences other than those of the pathogen of primary interest. It is preferable that such sequences are not of human origin, to minimize the risk of autoimmune disease. For RTS,S, one vaccine construct raises antibodies against HBV and malaria. Would pre-existing antibodies against the platform prevent a new vaccine providing protection? For RTS,S in a typical three dose treatment, booster doses do improve the malaria protection even though anti-HBsAg antibodies are already present (107). Similar results were found in mouse models with Spy-VLPs (39, 55, 81, 82). A recent human trial found that pre-existing cross-reactive antibodies from the inactivated Japanese encephalitis virus vaccine enhanced the response to subsequent live-attenuated yellow fever vaccination (108). Nonetheless, further testing of these questions will be important.

There is limited information whether SpyCatcher or SpyTag contains MHC class II epitopes, which might contribute to B cell memory. Deletion of the N-terminal region of SpyCatcher to give ΔN1SpyCatcher (92 aa, the construct used on AP205 by Brune et al.) reduced antibody titers against SpyCatcher (77). We showed that antibodies were raised to SpyCatcher-VLPs after immunization, but antibody levels were substantially reduced after conjugating SpyTag antigen (55).

## Summary and Future Opportunities

### Decoration Opportunities on Other Platforms

Assembly scaffolds other than non-enveloped VLPs have valuable properties for immune stimulation and could benefit from modular decoration with other antigens. Outer membrane vesicles (OMVs) are spherical membranous nanoparticles released from the outer membrane of Gram-negative bacteria. OMVs can be produced at large scale, displaying proteins of interest on their surface or packaging proteins stably inside. SpyTag was first used for OMV display through expression of a SpyTag-OmpA fusion, allowing conjugation of an enzyme for detoxification of nerve agents (109). SpyTag was subsequently displayed on hemoglobin protease, yielding OMVs (30–200 nm in diameter) from *E. coli* and *Salmonella Typhimurium* displaying SpyCatcher-linked pneumococcal antigens (24). In addition, this platform was amenable to SnoopTag/SnoopCatcher antigen display (24). The same authors also introduced the concept of attenuated, Spy-displaying live bacteria as vaccine carriers (24).

In the future, Tag/Catcher display may facilitate comparison between other non-protein vaccination platforms. Such platforms are beyond our scope to cover in detail, but include emulsions, inorganic NPs, immune-stimulating complexes, and lipid-based NPs (e.g., liposomes and spheroplasts) (4, 110, 111).

Virus-like particles are pre-eminent vaccine platforms for inducing antibody responses; a central challenge for the field is to develop platforms giving potent T cell and B cell responses, important for the most intractable pathogens such as tuberculosis or HIV (21, 112, 113). Though existing Catcher/Tag-VLNP platforms have not yet been thoroughly assessed for T cell responses, recent work has enhanced T cell induction based on co-delivered adjuvants (114), additional helper T-cell epitopes (115), or incorporating cell-permeable peptides (116).

Where VLNPs may not be able to provide a sufficient T cell response, viral platforms are leading the way at inducing cytotoxic T cell responses. SpyTag fusion has recently enabled modular modification of certain virus models, although immune stimulation has not been tested. The envelope protein GP64 from baculovirus was fused with SpyTag and labeled with SpyCatcher-labeled quantum dots for tracking and visualization in insect cells (117). SpyTag was also used in lentivirus targeting: target-cell tropism was redirected upon conjugating SpyTagged Sindbis virus E2 lentiviral envelope protein with SpyCatcher-linked anti-HER2-DARPin (118).

### Modularity Could Enhance VLP Vaccines for Chronic Diseases and Personalized Medicine

**Figure 4:**
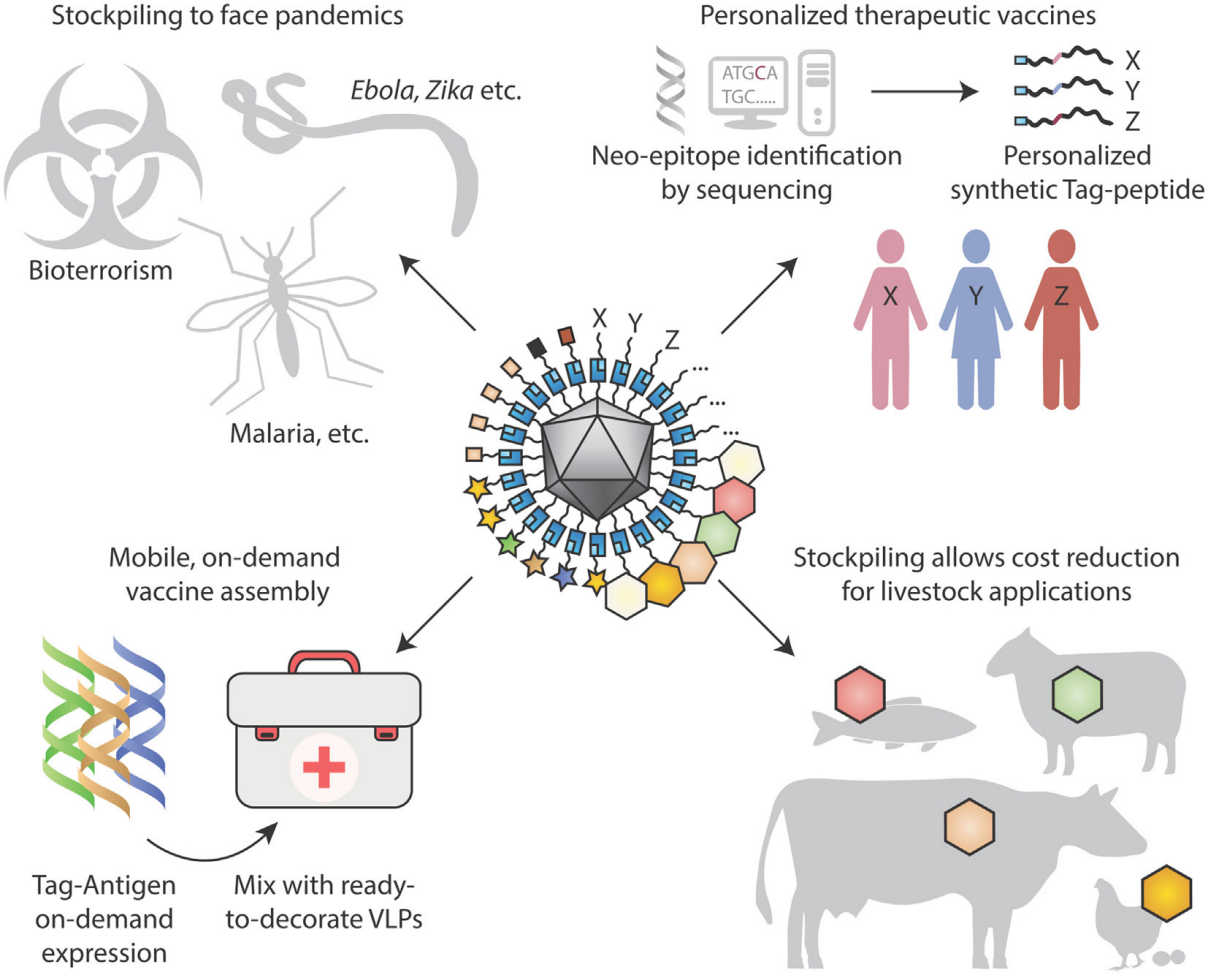
Looking to the future for modular vaccine assembly. (Top left) Stockpiling one underlying particulate scaffold against multiple diseases may facilitate cheap rapid production of vaccines, in the face of pandemics, bioterrorism, and tropical diseases. (Top right) Personalized therapeutic vaccines are coming into view through next-generation sequencing identifying cancer neo-epitopes. One may envision neo-epitopes being chemically synthesized and coupled specifically to a virus-like particle (VLP) scaffold for immunization. (Bottom left) On-site synthesis of Tag-antigens becomes possible with cell-free protein synthesis stable at ambient temperature. Subsequent coupling to pre-assembled virus-like nanoparticles may allow in-field vaccine generation, with minimal space and weight footprint for a large number of vaccine targets. (Bottom right) Stockpiling may drive down costs of VLP vaccines for animal health applications, to combat zoonoses, for pets and for agriculture.

Beyond infectious diseases, VLP platforms may be adopted for therapeutic use. Allergens on VLPs may suppress allergic immune responses, while avoiding side-effects from direct activation of mast cells (40). New therapeutic antibodies continue to achieve clinical success, including landmark results of checkpoint inhibitors against cancer (119). Rather than recombinantly expressing antibodies and then infusing, there may be advantages in inducing people to generate their own blocking antibodies (75, 80). Particulate vaccines presented here may be employed for non-infectious chronic diseases, such as Alzheimer’s disease or hypertension (120). With plummeting costs of DNA sequencing, personalized mutation-specific cancer therapy will advance rapidly (121), providing further drive for rapid assembly of personalized cancer vaccines (Figure 4).

In all fields of science, progress can come faster when one can move from patient crafting of success to a reproducible assembly line (122–126). Vaccines must satisfy many criteria, including efficacy, safety, manufacturability, and cost-effectiveness, so vaccinology will never be easy. But the gains in pathogen genomics (31) and structural vaccinology (17) can be complemented by the automation of antigen multimerization, as modular VLP decoration becomes routine.

## Author Contributions

KB and MH conceived and wrote the manuscript.

## Conflict of Interest Statement

KB is a consultant and co-founder of Genie Biotech Ltd. MH is an inventor on a patent concerning peptide targeting *via* spontaneous amide bond formation (EP2534484) and co-founded SpyBiotech Ltd.

## References

[1] Bachmann MF Jennings GT. Vaccine delivery: a matter of size, geometry, kinetics and molecular patterns. Nat Rev Immunol (2010) 10(11):787–96. 10.1038/nri2868 20948547

[2] Schiller J Lowy D. Explanations for the high potency of HPV prophylactic vaccines. Vaccine (2018). 10.1016/j.vaccine.2017.12.079 29325819 PMC6035892

[3] Karch CP Burkhard P. Vaccine technologies: from whole organisms to rationally designed protein assemblies. Biochem Pharmacol (2016) 120:1–14. 10.1016/j.bcp.2016.05.001 27157411 PMC5079805

[4] Gomes AC Mohsen M Bachmann MF Harnessing nanoparticles for immunomodulation and vaccines. Vaccines (Basel) (2017) 5(1):E6 10.3390/vaccines5010006 28216554 PMC5371742

[5] Cruz FM Colbert JD Merino E Kriegsman BA Rock KL. The biology and underlying mechanisms of cross-presentation of exogenous antigens on MHC-I molecules. Annu Rev Immunol (2017) 35:149–76. 10.1146/annurev-immunol-041015-055254 28125356 PMC5508990

[6] Huh WK Falvo JV Gerke LC Carroll AS Howson RW Weissman JS Global analysis of protein localization in budding yeast. Nature (2003) 425:686–91. 10.1038/nature02026 14562095

[7] Shishovs M Rumnieks J Diebolder C Jaudzems K Andreas LB Stanek J Structure of AP205 coat protein reveals circular permutation in ssRNA bacteriophages. J Mol Biol (2016) 428(21):4267–79. 10.1016/j.jmb.2016.08.025 27591890

[8] Walker A Skamel C Nassal M. SplitCore: an exceptionally versatile viral nanoparticle for native whole protein display regardless of 3D structure. Sci Rep (2011) 1:5. 10.1038/srep00005 22355524 PMC3216493

[9] Pejawar-Gaddy S Rajawat Y Hilioti Z Xue J Gaddy DF Finn OJ Generation of a tumor vaccine candidate based on conjugation of a MUC1 peptide to polyionic papillomavirus virus-like particles. Cancer Immunol Immunother (2010) 59(11):1685–96. 10.1007/s00262-010-0895-0 20652244 PMC3076734

[10] Stockley PG Twarock R Bakker SE Barker AM Borodavka A Dykeman E Packaging signals in single-stranded RNA viruses: nature’s alternative to a purely electrostatic assembly mechanism. J Biol Phys (2013) 39(2):277–87. 10.1007/s10867-013-9313-0 23704797 PMC3662417

[11] Zlotnick A. To build a virus capsid. An equilibrium model of the self assembly of polyhedral protein complexes. J Mol Biol (1994) 241(1):59–67. 10.1006/jmbi.1994.1473 8051707

[12] Kost TA Condreay JP Jarvis DL. Baculovirus as versatile vectors for protein expression in insect and mammalian cells. Nat Biotechnol (2005) 23(5):567–75. 10.1038/nbt1095 15877075 PMC3610534

[13] Liew MW Rajendran A Middelberg AP. Microbial production of virus-like particle vaccine protein at gram-per-litre levels. J Biotechnol (2010) 150(2):224–31. 10.1016/j.jbiotec.2010.08.010 20797415

[14] Lomonossoff GP D’Aoust MA. Plant-produced biopharmaceuticals: a case of technical developments driving clinical deployment. Science (2016) 353(6305):1237–40. 10.1126/science.aaf6638 27634524

[15] Nothaft H Szymanski CM. Bacterial protein N-glycosylation: new perspectives and applications. J Biol Chem (2013) 288(10):6912–20. 10.1074/jbc.R112.417857 23329827 PMC3591601

[16] Plotkin S Robinson JM Cunningham G Iqbal R Larsen S. The complexity and cost of vaccine manufacturing – an overview. Vaccine (2017) 35(33):4064–71. 10.1016/j.vaccine.2017.06.003 28647170 PMC5518734

[17] Dormitzer PR Grandi G Rappuoli R. Structural vaccinology starts to deliver. Nat Rev Microbiol (2012) 10(12):807–13. 10.1038/nrmicro2893 23154260

[18] Cullen LM Schmidt MR Morrison TG. The importance of RSV F protein conformation in VLPs in stimulation of neutralizing antibody titers in mice previously infected with RSV. Hum Vaccin Immunother (2017) 13:1–10. 10.1080/21645515.2017.1329069 28604155 PMC5718826

[19] Jardetzky T HIV: conformational camouflage. Nature (2002) 420(6916):623–4. 10.1038/420623a 12478278

[20] Burton DR Poignard P Stanfield RL Wilson IA. Broadly neutralizing antibodies present new prospects to counter highly antigenically diverse viruses. Science (2012) 337(6091):183–6. 10.1126/science.1225416 22798606 PMC3600854

[21] Burton DR Hangartner L. Broadly neutralizing antibodies to HIV and their role in vaccine design. Annu Rev Immunol (2016) 34:635–59. 10.1146/annurev-immunol-041015-055515 27168247 PMC6034635

[22] Kanekiyo M Wei CJ Yassine HM McTamney PM Boyington JC Whittle JR Self-assembling influenza nanoparticle vaccines elicit broadly neutralizing H1N1 antibodies. Nature (2013) 499(7456):102–6. 10.1038/nature12202 23698367 PMC8312026

[23] Brown SD Fiedler JD Finn MG. Assembly of hybrid bacteriophage Qbeta virus-like particles. Biochemistry (2009) 48(47):11155–7. 10.1021/bi901306p 19848414 PMC2799296

[24] van den Berg van Saparoea HB Houben D de Jonge MI Jong WSP Luirink J Display of recombinant proteins on bacterial outer membrane vesicles using protein ligation. Appl Environ Microbiol (2018) 84(8):e2567–2517. 10.1128/AEM.02567-17 PMC5881043 29439988

[25] Smith ML Fitzmaurice WP Turpen TH Palmer KE. Display of peptides on the surface of tobacco mosaic virus particles. Curr Top Microbiol Immunol (2009) 332:13–31. 10.1007/978-3-540-70868-1_2 19401819 PMC7122513

[26] Gedvilaite A Kucinskaite-Kodze I Lasickiene R Timinskas A Vaitiekaite A Ziogiene D Evaluation of trichodysplasia spinulosa-associated polyomavirus capsid protein as a new carrier for construction of chimeric virus-like particles harboring foreign epitopes. Viruses (2015) 7(8):4204–29. 10.3390/v7082818 26230706 PMC4576179

[27] McComb RC Ho CL Bradley KA Grill LK Martchenko M. Presentation of peptides from *Bacillus anthracis* protective antigen on tobacco mosaic virus as an epitope targeted anthrax vaccine. Vaccine (2015) 33(48):6745–51. 10.1016/j.vaccine.2015.10.075 26514421

[28] Geng F Suharlim C Brenzel L Resch SC Menzies NA. The cost structure of routine infant immunization services: a systematic analysis of six countries. Health Policy Plan (2017) 32:1174–84. 10.1093/heapol/czx067 28575193 PMC5886070

[29] Ghunaim H Desin TS. Potential impact of food safety vaccines on health care costs. Foodborne Pathog Dis (2015) 12(9):733–40. 10.1089/fpd.2014.1924 26111256

[30] Charlton Hume HK Lua LHL. Platform technologies for modern vaccine manufacturing. Vaccine (2017) 35(35 Pt A):4480–5. 10.1016/j.vaccine.2017.02.069 28347504 PMC7115529

[31] Proietti C Doolan DL The case for a rational genome-based vaccine against malaria. Front Microbiol (2014) 5:741 10.3389/fmicb.2014.00741 25657640 PMC4302942

[32] Crosnier C Wanaguru M McDade B Osier FH Marsh K Rayner JC A library of functional recombinant cell-surface and secreted *P. falciparum* merozoite proteins. Mol Cell Proteomics (2013) 12(12):3976–86. 10.1074/mcp.O113.028357 24043421 PMC3861738

[33] Pitoiset F Vazquez T Bellier B. Enveloped virus-like particle platforms: vaccines of the future? Expert Rev Vaccines (2015) 14(7):913–5. 10.1586/14760584.2015.1046440 25968245

[34] Pardee K Slomovic S Nguyen PQ Lee JW Donghia N Burrill D Portable, on-demand biomolecular manufacturing. Cell (2016) 167(1):248–59.e12. 10.1016/j.cell.2016.09.013 27662092

[35] Graham BS Sullivan NJ. Emerging viral diseases from a vaccinology perspective: preparing for the next pandemic. Nat Immunol (2018) 19(1):20–8. 10.1038/s41590-017-0007-9 29199281 PMC7097586

[36] O’Dowd A Government says it would stockpile Tamiflu again. BMJ (2014) 349:g6386 10.1136/bmj.g6386 25338644

[37] Desai SN Pezzoli L Martin S Costa A Rodriguez C Legros D A second affordable oral cholera vaccine: implications for the global vaccine stockpile. Lancet Glob Health (2016) 4(4):e223–4. 10.1016/S2214-109X(16)00037-1 27013303

[38] Yen C Hyde TB Costa AJ Fernandez K Tam JS Hugonnet S The development of global vaccine stockpiles. Lancet Infect Dis (2015) 15(3):340–7. 10.1016/S1473-3099(14)70999-5 25661473 PMC4712379

[39] Leneghan DB Miura K Taylor IJ Li Y Jin J Brune KD Nanoassembly routes stimulate conflicting antibody quantity and quality for transmission-blocking malaria vaccines. Sci Rep (2017) 7(1):3811. 10.1038/s41598-017-03798-3 28630474 PMC5476561

[40] Engeroff P Caviezel F Storni F Thoms F Vogel M Bachmann MF Allergens displayed on virus-like particles are highly immunogenic but fail to activate human mast cells. Allergy (2017) 73:341–9. 10.1111/all.13268 28787769

[41] Cavelti-Weder C Timper K Seelig E Keller C Osranek M Lassing U Development of an interleukin-1beta vaccine in patients with type 2 diabetes. Mol Ther (2016) 24(5):1003–12. 10.1038/mt.2015.227 26686385 PMC4881764

[42] Cornuz J Zwahlen S Jungi WF Osterwalder J Klingler K van Melle G A vaccine against nicotine for smoking cessation: a randomized controlled trial. PLoS One (2008) 3(6):e2547 10.1371/journal.pone.0002547 18575629 PMC2432028

[43] Strable E Finn MG. Chemical modification of viruses and virus-like particles. In: Manchester M Steinmetz NF, editors. Viruses and Nanotechnology. Berlin, Heidelberg: Springer, Berlin, Heidelberg (2009). p. 1–21. 10.1007/978-3-540-69379-6_1 19198568

[44] Stephanopoulos N Francis MB. Choosing an effective protein bioconjugation strategy. Nat Chem Biol (2011) 7(12):876–84. 10.1038/nchembio.720 22086289

[45] Koho T Ihalainen TO Stark M Uusi-Kerttula H Wieneke R Rahikainen R His-tagged norovirus-like particles: a versatile platform for cellular delivery and surface display. Eur J Pharm Biopharm (2015) 96:22–31. 10.1016/j.ejpb.2015.07.002 26170162

[46] Guignet EG Hovius R Vogel H. Reversible site-selective labeling of membrane proteins in live cells. Nat Biotechnol (2004) 22(4):440–4. 10.1038/nbt954 15034592

[47] Fairhead M Krndija D Lowe ED Howarth M. Plug-and-play pairing via defined divalent streptavidins. J Mol Biol (2014) 426(1):199–214. 10.1016/j.jmb.2013.09.016 24056174 PMC4047826

[48] Wu S-C Wong S-L. Engineering soluble monomeric streptavidin with reversible biotin binding capability. J Biol Chem (2005) 280(24):23225–31. 10.1074/jbc.M501733200 15840576

[49] Thrane S Janitzek CM Agerbaek MO Ditlev SB Resende M Nielsen MA A novel virus-like particle based vaccine platform displaying the placental malaria antigen VAR2CSA. PLoS One (2015) 10(11):e0143071. 10.1371/journal.pone.0143071 26599509 PMC4657905

[50] Lim KH Huang H Pralle A Park S. Stable, high-affinity streptavidin monomer for protein labeling and monovalent biotin detection. Biotechnol Bioeng (2013) 110(1):57–67. 10.1002/bit.24605 22806584

[51] Chackerian B Lowy DR Schiller JT. Conjugation of a self-antigen to papillomavirus-like particles allows for efficient induction of protective autoantibodies. J Clin Invest (2001) 108(3):415–23. 10.1172/JCI11849 11489935 PMC209354

[52] Leblanc P Moise L Luza C Chantaralawan K Lezeau L Yuan J VaxCelerate II: rapid development of a self-assembling vaccine for Lassa fever. Hum Vaccin Immunother (2014) 10(10):3022–38. 10.4161/hv.34413 25483693 PMC5443105

[53] Chivers CE Koner AL Lowe ED Howarth M. How the biotin-streptavidin interaction was made even stronger: investigation via crystallography and a chimaeric tetramer. Biochem J (2011) 435(1):55–63. 10.1042/BJ20101593 21241253 PMC3062853

[54] Venter PA Dirksen A Thomas D Manchester M Dawson PE Schneemann A. Multivalent display of proteins on viral nanoparticles using molecular recognition and chemical ligation strategies. Biomacromolecules (2011) 12(6):2293–301. 10.1021/bm200369e 21545187 PMC3114102

[55] Brune K Leneghan D Brian IJ Ishizuka AS Bachmann MF Draper SJ Plug-and-display: decoration of virus-like particles via isopeptide bonds for modular immunization. Sci Rep (2016) 6:19234. 10.1038/srep19234 26781591 PMC4725971

[56] Patel KG Swartz JR. Surface functionalization of virus-like particles by direct conjugation using azide-alkyne click chemistry. Bioconjug Chem (2011) 22(3):376–87. 10.1021/bc100367u 21355575 PMC5437849

[57] Aerni HR Shifman MA Rogulina S O’Donoghue P Rinehart J. Revealing the amino acid composition of proteins within an expanded genetic code. Nucleic Acids Res (2015) 43(2):e8. 10.1093/nar/gku1087 25378305 PMC4333366

[58] Sasmal PK Carregal-Romero S Han AA Streu CN Lin Z Namikawa K Catalytic azide reduction in biological environments. Chembiochem (2012) 13(8):1116–20. 10.1002/cbic.201100719 22514188

[59] Versteegen RM Rossin R ten Hoeve W Janssen HM Robillard MS Click to release: instantaneous doxorubicin elimination upon tetrazine ligation. Angew Chem Int Ed Engl (2013) 52(52):14112–6. 10.1002/anie.201305969 24281986

[60] Sun Q Chen Q Blackstock D Chen W. Post-translational modification of bionanoparticles as a modular platform for biosensor assembly. ACS Nano (2015) 9(8):8554–61. 10.1021/acsnano.5b03688 26235232

[61] Mazzucchelli S Colombo M Verderio P Rozek E Andreata F Galbiati E Orientation-controlled conjugation of haloalkane dehalogenase fused homing peptides to multifunctional nanoparticles for the specific recognition of cancer cells. Angew Chem Int Ed Engl (2013) 52(11):3121–5. 10.1002/anie.201209662 23386453

[62] Liu DS Phipps WS Loh KH Howarth M Ting AY. Quantum dot targeting with lipoic acid ligase and HaloTag for single-molecule imaging on living cells. ACS Nano (2012) 6(12):11080–7. 10.1021/nn304793z 23181687 PMC3528850

[63] Colombo M Mazzucchelli S Montenegro JM Galbiati E Corsi F Parak WJ Protein oriented ligation on nanoparticles exploiting O6-alkylguanine-DNA transferase (SNAP) genetically encoded fusion. Small (2012) 8(10):1492–7. 10.1002/smll.201102284 22431243

[64] Eckhardt M Anders M Muranyi W Heilemann M Krijnse-Locker J Muller B A SNAP-tagged derivative of HIV-1 – a versatile tool to study virus-cell interactions. PLoS One (2011) 6(7):e22007 10.1371/journal.pone.0022007 21799764 PMC3142126

[65] Antos JM Truttmann MC Ploegh HL. Recent advances in sortase-catalyzed ligation methodology. Curr Opin Struct Biol (2016) 38:111–8. 10.1016/j.sbi.2016.05.021 27318815 PMC5010448

[66] Tang S Xuan B Ye X Huang Z Qian Z. A modular vaccine development platform based on sortase-mediated site-specific tagging of antigens onto virus-like particles. Sci Rep (2016) 6:25741. 10.1038/srep25741 27170066 PMC4864371

[67] Chen Q Sun Q Molino NM Wang SW Boder ET Chen W. Sortase A-mediated multi-functionalization of protein nanoparticles. Chem Commun (Camb) (2015) 51(60):12107–10. 10.1039/c5cc03769g 26120946

[68] Shah NH Muir TW. Inteins: nature’s gift to protein chemists. Chem Sci (2014) 5(1):446–61. 10.1039/C3SC52951G 24634716 PMC3949740

[69] Muik A Reul J Friedel T Muth A Hartmann KP Schneider IC Covalent coupling of high-affinity ligands to the surface of viral vector particles by protein trans-splicing mediates cell type-specific gene transfer. Biomaterials (2017) 144:84–94. 10.1016/j.biomaterials.2017.07.032 28825979

[70] Lilie H Richter S Bergelt S Frost S Gehle F. Polyionic and cysteine-containing fusion peptides as versatile protein tags. Biol Chem (2013) 394(8):995–1004. 10.1515/hsz-2013-0116 23629522

[71] Stubenrauch K Gleiter S Brinkmann U Rudolph R Lilie H. Conjugation of an antibody Fv fragment to a virus coat protein: cell-specific targeting of recombinant polyoma-virus-like particles. Biochem J (2001) 356(Pt 3):867–73. 10.1042/0264-6021:3560867 11389696 PMC1221915

[72] Reddington SC Howarth M. Secrets of a covalent interaction for biomaterials and biotechnology: SpyTag and SpyCatcher. Curr Opin Chem Biol (2015) 29:94–9. 10.1016/j.cbpa.2015.10.002 26517567

[73] Zakeri B Fierer JO Celik E Chittock EC Schwarz-Linek U Moy VT Peptide tag forming a rapid covalent bond to a protein, through engineering a bacterial adhesin. Proc Natl Acad Sci U S A (2012) 109(12):E690–7. 10.1073/pnas.1115485109 22366317 PMC3311370

[74] Veggiani G Nakamura T Brenner MD Gayet RV Yan J Robinson CV Programmable polyproteams built using twin peptide superglues. Proc Natl Acad Sci U S A (2016) 113(5):1202–7. 10.1073/pnas.1519214113 26787909 PMC4747704

[75] Thrane S Janitzek CM Matondo S Resende M Gustavsson T de Jongh WA Bacterial superglue enables easy development of efficient virus-like particle based vaccines. J Nanobiotechnology (2016) 14:30. 10.1186/s12951-016-0181-1 27117585 PMC4847360

[76] Fairhead M Veggiani G Lever M Yan J Mesner D Robinson CV SpyAvidin hubs enable precise and ultrastable orthogonal nanoassembly. J Am Chem Soc (2014) 136(35):12355–63. 10.1021/ja505584f 25111182 PMC4183622

[77] Liu Z Zhou H Wang W Tan W Fu YX Zhu M. A novel method for synthetic vaccine construction based on protein assembly. Sci Rep (2014) 4:7266. 10.1038/srep07266 25434527 PMC4248271

[78] Liu Z Zhou H Wang W Fu YX Zhu M. A novel dendritic cell targeting HPV16 E7 synthetic vaccine in combination with PD-L1 blockade elicits therapeutic antitumor immunity in mice. Oncoimmunology (2016) 5(6):e1147641. 10.1080/2162402X.2016.1147641 27471615 PMC4938372

[79] Li L Fierer JO Rapoport TA Howarth M. Structural analysis and optimization of the covalent association between SpyCatcher and a peptide Tag. J Mol Biol (2014) 426(2):309–17. 10.1016/j.jmb.2013.10.021 24161952 PMC3959856

[80] Palladini A Thrane S Janitzek CM Pihl J Clemmensen SB de Jongh WA Virus-like particle display of HER2 induces potent anti-cancer responses. Oncoimmunology (2018) 7(3):e1408749. 10.1080/2162402X.2017.1408749 29399414 PMC5790387

[81] Janitzek CM Matondo S Thrane S Nielsen MA Kavishe R Mwakalinga SB Bacterial superglue generates a full-length circumsporozoite protein virus-like particle vaccine capable of inducing high and durable antibody responses. Malar J (2016) 15(1):545. 10.1186/s12936-016-1574-1 27825348 PMC5101663

[82] Brune KD Buldun CM Li Y Taylor IJ Brod F Biswas S Dual plug-and-display synthetic assembly using orthogonal reactive proteins for twin antigen immunization. Bioconjug Chem (2017) 28(5):1544–51. 10.1021/acs.bioconjchem.7b00174 28437083

[83] Singh SK Thrane S Janitzek CM Nielsen MA Theander TG Theisen M Improving the malaria transmission-blocking activity of a *Plasmodium falciparum* 48/45 based vaccine antigen by SpyTag/SpyCatcher mediated virus-like display. Vaccine (2017) 35(30):3726–32. 10.1016/j.vaccine.2017.05.054 28578824

[84] Matondo S Thrane S Janitzek CM Kavishe RA Mwakalinga SB Theander TG A VAR2CSA:CSP conjugate capable of inducing dual specificity antibody responses. Afr Health Sci (2017) 17(2):373–81. 10.4314/ahs.v17i2.11 29062332 PMC5637022

[85] Bonnet J Cartannaz J Tourcier G Contreras-Martel C Kleman JP Morlot C Autocatalytic association of proteins by covalent bond formation: a Bio Molecular Welding toolbox derived from a bacterial adhesin. Sci Rep (2017) 7:43564. 10.1038/srep43564 28252635 PMC5333627

[86] Schlick TL Ding Z Kovacs EW Francis MB. Dual-surface modification of the tobacco mosaic virus. J Am Chem Soc (2005) 127(11):3718–23. 10.1021/ja046239n 15771505

[87] Wang C Zhu W Wang BZ. Dual-linker gold nanoparticles as adjuvanting carriers for multivalent display of recombinant influenza hemagglutinin trimers and flagellin improve the immunological responses in vivo and in vitro. Int J Nanomedicine (2017) 12:4747–62. 10.2147/IJN.S137222 28740382 PMC5503497

[88] Hu S Xie Z Onishi A Yu X Jiang L Lin J Profiling the human protein-DNA interactome reveals ERK2 as a transcriptional repressor of interferon signaling. Cell (2009) 139(3):610–22. 10.1016/j.cell.2009.08.037 19879846 PMC2774939

[89] Gilbert C Howarth M Harwood CR Ellis T. Extracellular self-assembly of functional and tunable protein conjugates from *Bacillus subtilis*. ACS Synth Biol (2017) 6(6):957–67. 10.1021/acssynbio.6b00292 28230977

[90] Singh SK Roeffen W Mistarz UH Chourasia BK Yang F Rand KD Construct design, production, and characterization of *Plasmodium falciparum* 48/45 R0.6C subunit protein produced in *Lactococcus lactis* as candidate vaccine. Microb Cell Fact (2017) 16(1):97. 10.1186/s12934-017-0710-0 28569168 PMC5452637

[91] Roder J Fischer R Commandeur U. Engineering potato virus X particles for a covalent protein based attachment of enzymes. Small (2017) 13(48):1702151. 10.1002/smll.201702151 29125698

[92] Hinrichsen M Lenz M Edwards JM Miller OK Mochrie SGJ Swain PS A new method for post-translationally labeling proteins in live cells for fluorescence imaging and tracking. Protein Eng Des Sel (2017) 30(12):771–80. 10.1093/protein/gzx059 29228311 PMC6680098

[93] Lee BY Wedlock PT Haidari LA Elder K Potet J Manring R Economic impact of thermostable vaccines. Vaccine (2017) 35(23):3135–42. 10.1016/j.vaccine.2017.03.081 28455169 PMC5547751

[94] Qi X Xiong S. Intein-mediated backbone cyclization of VP1 protein enhanced protection of CVB3-induced viral myocarditis. Sci Rep (2017) 7:41485. 10.1038/srep41485 28148910 PMC5288654

[95] Scheiblhofer S Laimer J Machado Y Weiss R Thalhamer J. Influence of protein fold stability on immunogenicity and its implications for vaccine design. Expert Rev Vaccines (2017) 16(5):479–89. 10.1080/14760584.2017.1306441 28290225 PMC5490637

[96] Schoene C Bennett SP Howarth M. SpyRing interrogation: analyzing how enzyme resilience can be achieved with phytase and distinct cyclization chemistries. Sci Rep (2016) 6:21151. 10.1038/srep21151 26861173 PMC4748275

[97] Giessen TW Silver PA. A catalytic nanoreactor based on in vivo encapsulation of multiple enzymes in an engineered protein nanocompartment. Chembiochem (2016) 17(20):1931–5. 10.1002/cbic.201600431 27504846

[98] Milburn MV Hassell AM Lambert MH Jordan SR Proudfoot AE Graber P A novel dimer configuration revealed by the crystal structure at 2.4 A resolution of human interleukin-5. Nature (1993) 363(6425):172–6. 10.1038/363172a0 8483502

[99] Engelsma D Rodriguez JA Fish A Giaccone G Fornerod M. Homodimerization antagonizes nuclear export of survivin. Traffic (2007) 8(11):1495–502. 10.1111/j.1600-0854.2007.00629.x 17714426

[100] Jennings GT Bachmann MF. The coming of age of virus-like particle vaccines. Biol Chem (2008) 389(5):521–36. 10.1515/BC.2008.064 18953718

[101] Patel JM Vartabedian VF Kim MC He S Kang SM Selvaraj P. Influenza virus-like particles engineered by protein transfer with tumor-associated antigens induces protective antitumor immunity. Biotechnol Bioeng (2015) 112(6):1102–10. 10.1002/bit.25537 25689082 PMC4621003

[102] Spohn G Jennings GT Martina BE Keller I Beck M Pumpens P A VLP-based vaccine targeting domain III of the West Nile virus E protein protects from lethal infection in mice. Virol J (2010) 7:146. 10.1186/1743-422X-7-146 20604940 PMC2914671

[103] Keeble AH Banerjee A Reddington SC Ferla MP Howarth M Khairil Anuar INA. Evolving accelerated amidation by SpyTag/SpyCatcher to analyze membrane dynamics. Angew Chem Int Ed Engl (2017) 56(52):16521–5. 10.1002/anie.201707623 29024296 PMC5814910

[104] Zakeri B Howarth M. Spontaneous intermolecular amide bond formation between side chains for irreversible peptide targeting. J Am Chem Soc (2010) 132(13):4526–7. 10.1021/ja910795a 20235501

[105] Cao Y Liu D Zhang WB. Supercharging SpyCatcher toward an intrinsically disordered protein with stimuli-responsive chemical reactivity. Chem Commun (Camb) (2017) 53(63):8830–3. 10.1039/c7cc04507g 28692103

[106] Proschel M Kraner ME Horn AHC Schafer L Sonnewald U Sticht H. Probing the potential of CnaB-type domains for the design of tag/catcher systems. PLoS One (2017) 12(6):e0179740. 10.1371/journal.pone.0179740 28654665 PMC5487036

[107] RTS,S Clinical Trials Partnership Agnandji ST Lell B Fernandes JF Abossolo BP Methogo BG A phase 3 trial of RTS,S/AS01 malaria vaccine in African infants. N Engl J Med (2012) 367(24):2284–95. 10.1056/NEJMoa1208394 23136909 PMC10915853

[108] Chan KR Wang X Saron WA Gan ES Tan HC Mok DZ Cross-reactive antibodies enhance live attenuated virus infection for increased immunogenicity. Nat Microbiol (2016) 1:16164. 10.1038/nmicrobiol.2016.164 27642668 PMC7097525

[109] Alves NJ Turner KB Daniele MA Oh E Medintz IL Walper SA Bacterial nanobioreactors – directing enzyme packaging into bacterial outer membrane vesicles. ACS Appl Mater Interfaces (2015) 7(44):24963–72. 10.1021/acsami.5b08811 26479678

[110] Schwendener RA. Liposomes as vaccine delivery systems: a review of the recent advances. Ther Adv Vaccines (2014) 2(6):159–82. 10.1177/2051013614541440 25364509 PMC4212474

[111] Ansari MA Zia Q Kazmi S Ahmad E Azhar A Johnson KE Efficacy of cell wall-deficient spheroplasts against experimental murine listeriosis. Scand J Immunol (2015) 82(1):10–24. 10.1111/sji.12296 25833403

[112] Collins KA Snaith R Cottingham MG Gilbert SC Hill AVS. Enhancing protective immunity to malaria with a highly immunogenic virus-like particle vaccine. Sci Rep (2017) 7:46621. 10.1038/srep46621 28422178 PMC5395940

[113] De Gregorio E Rappuoli R. From empiricism to rational design: a personal perspective of the evolution of vaccine development. Nat Rev Immunol (2014) 14(7):505–14. 10.1038/nri3694 24925139 PMC7096907

[114] Gomes AC Flace A Saudan P Zabel F Cabral-Miranda G Turabi AE Adjusted particle size eliminates the need of linkage of antigen and adjuvants for appropriated T cell responses in virus-like particle-based vaccines. Front Immunol (2017) 8:226. 10.3389/fimmu.2017.00226 28321220 PMC5337491

[115] Zeltins A West J Zabel F El Turabi A Balke I Haas S Incorporation of tetanus-epitope into virus-like particles achieves vaccine responses even in older recipients in models of psoriasis, Alzheimer’s and cat allergy. NPJ Vaccines (2017) 2:30. 10.1038/s41541-017-0030-8 29263885 PMC5653761

[116] Akhras S Toda M Boller K Himmelsbach K Elgner F Biehl M Cell-permeable capsids as universal antigen carrier for the induction of an antigen-specific CD8(+) T-cell response. Sci Rep (2017) 7(1):9630 10.1038/s41598-017-08787-0 28851900 PMC5575276

[117] Ke X Zhang Y Zheng F Liu Y Zheng Z Xu Y SpyCatcher-SpyTag mediated in situ labelling of progeny baculovirus with quantum dots for tracking viral infection in living cells. Chem Commun (Camb) (2018) 54(10):1189–92. 10.1039/c7cc08880a 29334085

[118] Kasaraneni N Chamoun-Emanuelli AM Wright G Chen Z. Retargeting lentiviruses via SpyCatcher-SpyTag chemistry for gene delivery into specific cell types. MBio (2017) 8(6):e1860–1817. 10.1128/mBio.01860-17 29233896 PMC5727413

[119] Sharma P Immune checkpoint therapy and the search for predictive biomarkers. Cancer J (2016) 22(2):68–72. 10.1097/PPO.0000000000000185 27111900 PMC4847150

[120] Chackerian B Frietze KM Moving towards a new class of vaccines for non-infectious chronic diseases. Expert Rev Vaccines (2016) 15(5):561–3. 10.1586/14760584.2016.1159136 26919571

[121] Bethune MT Joglekar AV. Personalized T cell-mediated cancer immunotherapy: progress and challenges. Curr Opin Biotechnol (2017) 48:142–52. 10.1016/j.copbio.2017.03.024 28494274

[122] Gross BC Erkal JL Lockwood SY Chen C Spence DM. Evaluation of 3D printing and its potential impact on biotechnology and the chemical sciences. Anal Chem (2014) 86(7):3240–53. 10.1021/ac403397r 24432804

[123] Kolb HC Finn MG Sharpless KB. Click chemistry: diverse chemical function from a few good reactions. Angew Chem Int Ed Engl (2001) 40(11):2004–21. 10.1002/1521-3773(20010601)40:11<2004::AID-ANIE2004>3.0.CO;2-5 11433435

[124] Terwilliger TC Stuart D Yokoyama S. Lessons from structural genomics. Annu Rev Biophys (2009) 38:371–83. 10.1146/annurev.biophys.050708.133740 19416074 PMC2847842

[125] Way JC Collins JJ Keasling JD Silver PA. Integrating biological redesign: where synthetic biology came from and where it needs to go. Cell (2014) 157(1):151–61. 10.1016/j.cell.2014.02.039 24679533

[126] Seeberger PH. The logic of automated glycan assembly. Acc Chem Res (2015) 48(5):1450–63. 10.1021/ar5004362 25871824

